# Impaired STAT3-Dependent Upregulation of IL2Rα in B Cells of a Patient With a STAT1 Gain-of-Function Mutation

**DOI:** 10.3389/fimmu.2019.00768

**Published:** 2019-04-24

**Authors:** Menno C. van Zelm, Julian J. Bosco, Pei M. Aui, Samuel De Jong, Fiona Hore-Lacy, Robyn E. O'Hehir, Robert G. Stirling, Paul U. Cameron

**Affiliations:** ^1^Department of Immunology and Pathology, Central Clinical School, Monash University, Melbourne, VIC, Australia; ^2^Allergy, Asthma and Clinical Immunology Service, Department of Respiratory, Allergy and Clinical Immunology (Research), Central Clinical School, Monash University, The Alfred Hospital, Melbourne, VIC, Australia; ^3^The Jeffrey Modell Diagnostic and Research Centre for Primary Immunodeficiencies in Melbourne, Melbourne, VIC, Australia; ^4^Department of Infectious Diseases, Alfred Hospital and Monash University, Melbourne, VIC, Australia; ^5^The Peter Doherty Institute for Infection and Immunity, University of Melbourne and Royal Melbourne Hospital, Melbourne, VIC, Australia

**Keywords:** chronic mucocutaneous candidiasis, hypogammaglobulinemia, STAT1, gain-of-function, STAT3, IL2Rα

## Abstract

Heterozygous *STAT1* gain-of-function (GOF) mutations form the most common genetic cause of chronic mucocutaneous candidiasis (CMC). In such patients, increased STAT1 function leads to impaired STAT3-dependent activation of IL-17A and IL-17F in T cells, thereby causing impaired Th17 responses to *Candida*. In spite of the critical role of STAT3 in IL-21 signaling in B cells, nearly all STAT1 GOF patients have normal or high serum IgG. We here present a 44 year-old male with childhood onset of CMC and antibody deficiency since early adulthood. Sequence analysis of *STAT1* revealed a heterozygous missense mutation in the coiled-coil domain (p.D168E), which resulted in increased STAT1 phosphorylation of B-cells activated with IFNα and IFNγ. IL-21 induced STAT3 phosphorylation and nuclear localization were normal, but resulted in impaired upregulation of IL2Rα. This newly identified B-cell intrinsic impairment of STAT3 function could underlie the progressive development of hypogammaglobulinemia. Considering the high risk of bronchiectasis and irreversible organ damage, this case illustrates the need for monitoring of IgG levels and/or function in adult patients with STAT1 GOF mutations.

## Background

Chronic mucocutaneous candidiasis (CMC) is a persistent or recurrent infection by *Candida* and typically affects the nails, skin, oral, and genital mucosae. In recent years, many cases have been shown to result from primary immunodeficiencies (PIDs) with impaired helper-T(h)17 cell immunity (1). This can be due to inhibitory autoantibodies against Th17 cytokines in patients with autosomal recessive (AR) polyendocrine syndrome type I (APS-1), or alternatively, inherited mutations that impair development and function of Th17 cells. Heterozygous *STAT1* gain-of-function (GOF) mutations form the most common genetic cause of CMC with mutations found in more than 50% of patients (2–4). These mutations are typically found in exons 7-14 which encode the coiled-coil and DNA-binding domains. As a result, increased STAT1 phosphorylation occurs upon stimulation of immune cells with STAT1-activating cytokines, such as interferon (IFN)α and IFNγ. Importantly, increased STAT1 signaling reciprocally inhibits STAT3-dependent cytokine production, which include IL-17A and IL-17F in T cells. Thus, STAT1 GOF predisposes to impaired Th17 responses to *Candida* (2, 4).

Patients with *STAT1* GOF mutations often present with additional bacterial and viral complications. Furthermore, autoimmunity/autoinflammatory disease has been observed in 37% of patients in a large cohort study (*n* = 274), and several patients have been shown to develop solid tumors (3). Effects on B-cells and humoral immunity are variable. 19% of 209 patients carried reduced total B cell numbers and 49% of the 53 patients examined had reduced memory B cell numbers. In addition, up to 23% of patients have impaired antibody responses to vaccinations with protein antigens, although only 3% have hypogammaglobulinemia (3, 5). As STAT3 is critical for IL21-dependent signaling in T-cell dependent B-cell responses, it is possible that STAT1 GOF mutations affect antibody responses and humoral immunity by inadvertent repression of STAT3-mediated transcription. We here identify a defect in STAT3-dependent upregulation of IL2Rα (CD25) in B cells of a patient with STAT1 GOF.

## Methods

### Ethics

Diagnostic work-up of blood and laboratory research studies including genetics of the patient were carried out with approval of Human Research Ethics committee of The Alfred Hospital (Study 109/15) and obtained after written informed consent. In addition, the patient has consented to publication of the case report. Data from healthy controls were collected after written consent was obtained and with approval of the human ethics committee of Monash University (Study 2016-0289). All studies were performed in accordance with the Declaration of Helsinki.

### Flowcytometric Immunophenotyping and *in vitro* Cell Stimulation

Patient and control subjects were included over a time period of 3 years. Standardized sample preparation, antibody staining, and flow cytometer instrument settings were used to ensure consistency in flow cytometry (6). In short, absolute counts of CD3+, CD4+ and CD8+ T cells, CD19+ B cells, and CD16+/CD56+ natural killer cells were obtained with a diagnostic lyse-no-wash protocol by using commercial Trucount tubes (BD Biosciences, San Jose, CA). For detailed 11-color flow cytometry, red blood cells were lysed with NH_4_Cl before incubation of 1–2 million nucleated cells for 15 min at room temperature in a total volume of 100 μL. After preparation, cells were measured on 4-laser flow cytometer (LSRII or LSRFortessa, BD Biosciences) by using standardized settings (6). Data were analyzed with FACSDiva (V8.0; BD Biosciences) and FlowJo software (v10) Naive and memory B-cells, and CD4+ T-cell subsets were defined as previously described (7).

Immortalization of patient's and control B cells with EBV derived from supernatant of the B95–8 cell line was performed as described previously (8). The EBV LCL were stimulated *in vitro* for 30 min with IFNα (10,000 U/ml; pbl assay science), IFN-γ (10,000 U/ml; Peprotech), or IL-21 (50 ng/ml; Lonza). Subsequently, the cells were stained with CD20-BV605 (clone 2H7; BioLegend) and Fixable Viability Stain 700 (BD Biosciences) prior to fixation, permeabilization, and staining with STAT1(pY701)-AF67 (clone 4a) and STAT3(pY705)-PE (clone 4/P-STAT3) according to manufacturer's instructions (BD Biosciences). Following acquisition on a 4-laser LSRII (BD Biosciences), live single cells that were positive for CD20 expression were analyzed for intracellular pSTAT1 and pSTAT3 expression (FlowJo v10). In addition, nuclear localization of pSTAT3 following 30 min IL-21 stimulation was determined in EBV LCL from the patient and from a healthy control using an imaging flow cytometer (Imagestream^X^ MKII; Amnis/Millennium Science, Mulgrave, VIC, Australia) equipped with four lasers (405, 488, 642, and 785 nm). Images (60x) were obtained from >1,000 cells per condition and similarity scores were derived for pSTAT3 and the nucleus (stained with Vybrant™ DyeCycle™ Violet; Thermo Scientific). Similarity scores for surface CD20 (BV605) and the nucleus were derived as negative control.

### Sequence Analysis of STAT1

Following genomic DNA isolation from post-Ficoll granulocytes (GenElute Mammalian Genomic DNA Miniprep Kit, Sigma-Aldrich, St Louis, Mo), exons 7-14 of the *STAT1* gene were PCR-amplified using previously published primers (9), and sequenced by the Micromon facility of Monash University on an Applied Biosystems 3730s DNA Analyzer (Thermo Fisher). Obtained sequences were aligned with the reference sequence from Ensembl using CLC Main Workbench 7 software.

### Molecular Analysis of Ig Gene Rearrangements

RNA was isolated from post-Ficoll mononuclear cells of the patient with a GenElute mammalian RNA kit (Sigma-Aldrich) and reverse transcribed to cDNA with random primers (Invitrogen Life technologies). Rearranged IgG and IgA transcripts were amplified in a multiplex PCR approach using 4 different *IGHV*-family leader forward primers in combination with an *IGHG*-consensus or *IGHA*-consensus reverse primer (10, 11). PCR products were cloned into a pGEMT easy vector (Promega, Madison WI), amplified by colony PCR, and sequenced as above. Sequences were analyzed using the IMGT database (http://www.imgt.org/IMGT_vquest/vquest) to assign the *IGHV, IGHD*, and *IGHJ* genes and alleles, and to identify somatic hypermutations (SHM). Of each unique clone, the position and frequency of mutations were determined within the entire *IGHV* gene (FR1-CDR1-FR2-CDR2-FR3). SHM were determined as variations on the best matched V-gene and represented as the percentage of mutations of the total sequenced V-gene nucleotides. The IgG and IgA receptor subclasses were determined using the *IGH* reference sequence (NG_001019). All results of the patient were compared with previously generated data sets of controls (12).

## Case Presentation

### Clinical History

We here present a 44 year-old male with a history of CMC treated since early childhood with azole antifungal agents. The patient is the second of three children from non-consanguineous parents. He has developed resistance to antifungal drugs including nystatin, fluconazole, and partially to voriconazole to which he had an allergic drug reaction of troublesome and persistent photodermatitis. He is currently controlled on posaconazole and amphotericin lozenges.

The CMC has been associated with the development of esophageal strictures requiring repeated dilation. At the age of 39 years this procedure was complicated by esophageal rupture and mediastinitis requiring a prolonged ICU admission. The esophageal rupture was treated surgically but subsequent investigations for recurrent stenosis led to diagnosis of esophageal cancer at age 40. He underwent esophageal resection a year later with clear surgical margins, followed by adjuvant chemotherapy which was truncated because of severe mucositis. Radiotherapy was commenced for this cancer due to poor prognosis in young age.

Shortly after diagnosis with esophageal cancer, the patient was started on G-CSF therapy (2 times 300 μg per week) for almost 2 years (Dec 2014–July 2016). As the patient reported increased discomfort following discontinuation, G-CSF therapy was re-started a year later at age 43 years and is still current.

During early adulthood, the patient developed progressive hypogammaglobulinemia (Table 1) with poor vaccine responses and commenced IVIG replacement at age 35. In spite of adequate trough IgG with monthly IVIG, he continues to suffer from recurrent lower respiratory tract infections requiring antibiotics and has been hospitalized on at least 4 occasions with bacterial infections, including salmonella gastroenteritis. He has required periodic courses of IV caspafungin for candida partially resistant to azoles.

**Table 1:** Immunological data.

**Laboratory measurement**	**Patient**		Normal range^*^
	**35 year**	**42 year**	
**SERUM IG LEVELS (g/L)**			
IgG	**3.4**	–	7.0–15.5
IgG1	**1.8**	–	3.8–9.3
IgG2	**1.8**	–	2.4–7.0
IgG3	0.3	–	0.22–1.76
IgG4	0.1	–	0.04–0.86
IgA	1.6	–	0.76–3.9
IgM	**0.3**	–	0.45–2.3
**LYMPHOCYTE SUBSETS (CELLS/μl BLOOD)**			
B cells	**51**	103	76–608
Transitional	–	0.9	0.4–29
Naive mature	–	87	31–398
IgD+ memory	–	7.1	3.4–79
IgD- memory	–	**2.4**	12–114
T cells	1,248	1,013	773–2,757
CD8	576	475	243–950
CD4	624	409	307–1,600
Tfh (CD45RA-CXCR5+)	–	26	16–175
Th17 (CD45RA-CCR6-CCR4+CXCR3-)	–	**7.4**	11–98

* *For cell subsets: 5–95% of adult controls; B cells, n = 44; T cells, n = 34. Values below normal range are depicted in bold font*.

### Identification of a Heterozygous Gain-of-Function Mutation in *STAT1*

**Figure 1:**
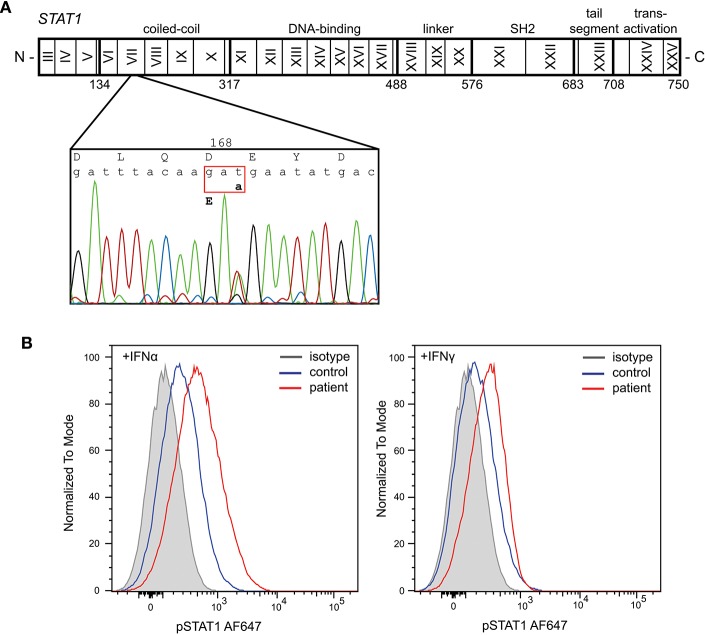
Identification of a heterozygous mutation in STAT1 leading to a gain of function. **(A)** Sanger sequencing revealed a heterozygous c.504T>A mutation in exon 7 resulting in a missense mutation in the coiled-coil domain (p.D168E). **(B)** Increased phosphorylation of STAT1 following *in vitro* stimulation of patient's EBV-LCL with IFNα and IFNγ.

Given the severity of the CMC and the antibody deficiency, more detailed immunological work-up was performed in the context of a research study. Detailed flowcytometric immunophenotyping of the patient's B- and T-cells revealed a severe reduction in CD27+ memory B cells and low circulating numbers of Th17 cells at age 42 years following discontinuation of G-CSF therapy (Table 1). As the patient did not have typical clinical associations of APS-1, a *STAT1* GOF mutation was considered and genetic analysis of *STAT1* exons 7-14 was performed on DNA of the patient. Sanger sequencing revealed a heterozygous variant in exon 7 (c.504T>A) resulting in a missense mutation in the coiled-coil domain (p.D168E) (Figure 1A). The same mutation has been previously described in a 5 year old female patient, but was not functionally addressed (3). To examine the effects of the mutation, we studied phosphorylation of STAT1 in EBV-immortalized B-lymphocytes of the patient. Thirty minutes after stimulation with either IFNα or IFNγ, the patient's cells showed increased levels of pSTAT1 confirming a GOF phenotype as a result of the D168E missense mutation (Figure 1B).

### T-Helper and T-Follicular Helper Cell Subsets

**Figure 2:**
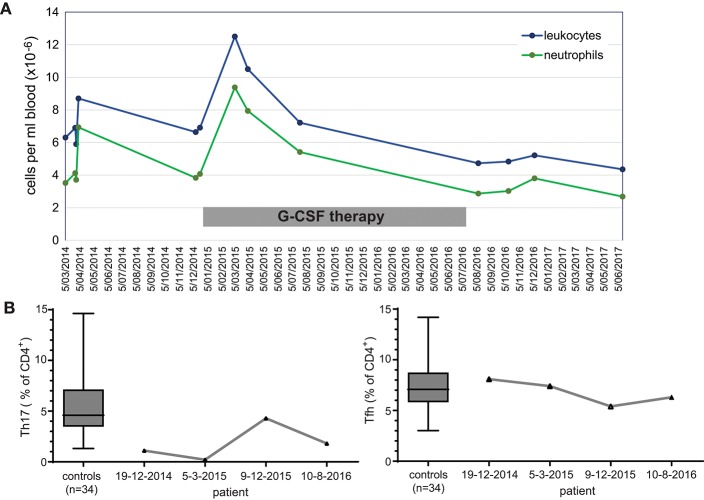
Immunological effects of immunotherapy with G-CSF. **(A)** Longitudinal measurements of blood leukocyte and neutrophil cell counts over a 3 year interval. **(B)** Frequencies of Th17 and Tfh cell subsets within total CD4 T cells prior to, during (2 timepoints; 2015) and after discontinuation of G-CSF therapy.

Given that the patient reported beneficial effects of G-CSF treatment, we retrospectively analyzed immune cells prior-to and during the treatment period. Extensive follow-up of total leukocyte and neutrophil count showed a general increase in numbers during therapy (Figure 2A). Three stored PBMC samples were available for detailed T-cell immunophenotyping, and reporting of relative frequencies of Th17 and Tfh cells. Th17 cell frequencies were within the normal range on only 1 occasion under G-CSF therapy, whereas Tfh cell frequencies were not below the normal range (Figure 2B). Hence, G-CSF therapy was associated with normalization of Th17 cells on at least one occasion.

### STAT3 Signaling Defect in B Cells

**Figure 3:**
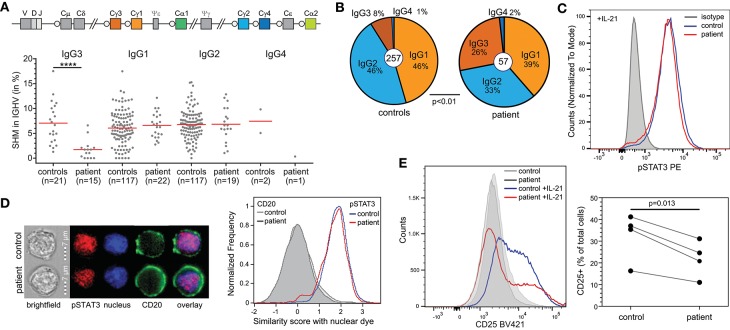
B-cell defects in STAT1 GOF. **(A)** Somatic hypermutation levels in IgG subclass transcripts of the patient and controls. **(B)** Relative distributions of IgG subclasses of unique transcripts. **(C)** STAT3 phosphorylation in EBV-LCL upon stimulation with IL-21. **(D)** Nuclear localization of pSTAT3 in EBV-LCL following stimulation with IL-21 as determined with the AMNIS ImageStream. Example images are shown on the left, and the collated events as overlays for nuclear localization scores on the right. **(E)** CD25 (IL2Rα) expression on EBV-LCL following stimulation with IL-21. Overlays are shown for one experiment to illustrate on the left and the combined data from 4 independent experiments on the right. Statistics: Paired *t*-test.

To gain more insight into the nature of the hypogammaglobulinemia and reduced memory B cells in the patient, we first quantified SHM in IgG transcripts from blood B cells. Overall, SHM levels were normal. However, IgG3 transcripts of the patient contained negligible SHM, in contrast to IgG1 and IgG2 (Figure 3A). Further analysis of the IgG transcripts demonstrated a predominant usage of IgG3 compared to IgG2 (Figure 3B).

T-cell dependent B-cell responses critically depend on IL-21R signaling via STAT3. As STAT1 GOF mutations can inhibit STAT3 activity, we here questioned whether the patient's B cells had intrinsically impaired STAT3 responsiveness. Indeed, in EBV-immortalized B cells from the patient, IL-21 stimulation normally induced STAT3 phosphorylation (Figure 3C). Moreover, nuclear localization studies with imaging flowcytometry revealed normal nuclear localization of pSTAT3 after IL-21 stimulation as well (Figure 3D). Therefore, we next evaluated functional STAT3 signaling by evaluation of expression of CD25, the IL2Rα chain, which is a direct target of STAT3 in B cells (13). Following 24 h incubation with IL-21, EBV-LCL from a healthy control upregulated CD25 surface expression (Figure 3E). In contrast, EBV-LCL from the patient had lower levels of CD25 expression. These findings are consistent with previous finding that STAT3 activity was inhibited by STAT1 GOF at the target gene activation level, but not upstream of that (14).

## Discussion

We here report a patient with STAT1 GOF and adult-onset antibody deficiency in the context of reduced total and memory B cells and impaired SHM and class switching to IgG2. Despite the known inhibition of STAT3 function due to STAT1 GOF, this has not been extensively addressed in previous studies. In contrast to the highly penetrant defects in Th17 function, the impact of *STAT1* GOF mutations on B-cell function and antibody responses is variable among reported patients (3). In B cells from our patient, we confirmed that following stimulation with IL-21, STAT3 phosphorylation and nuclear localization were not affected, but that activation of expression of the target gene encoding CD25 (IL-2Rα) was impaired. Hence, these B-cells will not be optimally sensitized to the stimulatory effects of IL-2 (13), and subject to suboptimal humoral immune responses.

Using *in vitro* functional analysis, we showed that the heterozygous *STAT1* D168E mutation in our patient had a dominant GOF effect on STAT1 phosphorylation. The increased amount of pSTAT1 protein following activation is generally assumed to be the result of a larger fraction of the mutant STAT1 being phosphorylated. However, as we were unable to measure total STAT1 protein, it remains possible that the mutant STAT1 is expressed at a higher level than the wild type, providing more total protein to be phosphorylated upon activation.

The same D168E mutation has been identified previously in a 5 year-old from Moroccan descent (patient 167 from kindred 111), but was not functionally assessed (3). The Moroccan girl did not present with hypogammaglobulinemia (IgG, 15.86 g/L), nor did our patient before early adulthood. It is therefore possible that patients with STAT1 GOF mutations are susceptible for a progressive decline in serum IgG levels and consequent antibody deficiency. This decline in antibody responses could be the accumulated effect of Tfh cell defects and the B-cell intrinsic defect to respond to IL-21. As shown by our *in vitro* data, this potentially mimics STAT3 LOF mutations with impaired activation of STAT3 target genes (13).

In this patient the STAT1 GOF may have directly contributed to the development of esophageal cancer. Early onset cancers were present in 6% of patients in the large series (3), and suggest that immunological abnormalities including Th17 and B cell function may also be associated with risk for malignancies.

Prior to the genetic diagnosis of STAT1 GOF, the patient was started on experimental G-CSF treatment based on the well-described anti candida function of this cytokine, as well as indications in the literature of a beneficial effect of adjunctive immunotherapy for the treatment of disseminated candidiasis (15). G-CSF therapy in our patient did increase Th17 cell frequencies, but did not resolve the candidiasis. The latter is in line with a recent report of an impaired capacity for killing of *C. albicans* by G-CSF recruited neutrophils (16). Moreover, it was previously reported that Th17 responses in STAT1 GOF patients were not restored by G-CSF immunotherapy (17). We here show a partial effect of G-CSF resulting in a clinical benefit. Increasing evidence is accumulating that Jak inhibitors are successful in resolving candidiasis in STAT1 GOF patients (18–20), but this is not entirely risk-free (21). Unfortunately, we have not yet been able to obtain compassionate access to Jak inhibitors for our patient, and as he does not experience adverse effects of G-CSF therapy this therapy is still continued.

## Concluding Remarks

We describe a B-cell intrinsic impairment of STAT3 function in a patient with a STAT1 GOF mutation and progressive development of hypogammaglobulinemia. Currently, a minority of the reported patients with STAT1 GOF mutations suffer from hypogammaglobulinemia with a larger proportion showing impaired responses to vaccination (3). Considering the high risk of bronchiectasis and irreversible organ damage (22, 23), this case illustrates the need for monitoring of IgG levels and/or function in adult patients with STAT1 GOF mutations.

## Ethics Statement

Diagnostic work-up of blood and laboratory research studies including genetics of the patient were carried out with approval of Human Research Ethics committee of The Alfred Hospital (Study 109/15) and obtained after written informed consent. Data from healthy controls were collected after written consent was obtained and with approval of the human ethics committee of Monash University (Study 2016-0289). All studies were performed in accordance with the Declaration of Helsinki.

## Author Contributions

MvZ, JB, and PC designed and wrote the manuscript. PA and SDJ performed experiments. FH-L, RO, and RS contributed to essential discussion of the paper. All authors critically read and commented on manuscript drafts and approved of the final version.

### Conflict of Interest Statement

The authors declare that the research was conducted in the absence of any commercial or financial relationships that could be construed as a potential conflict of interest.
